# Dominant Elongase Activity of *Elovl5a* but Higher Expression of *Elovl5b* in Common Carp (*Cyprinus carpio*)

**DOI:** 10.3390/ijms232314666

**Published:** 2022-11-24

**Authors:** Ran Zhao, Ya-Xin Wang, Chen-Ru Yang, Shang-Qi Li, Jin-Cheng Li, Xiao-Qing Sun, Hong-Wei Wang, Qi Wang, Yan Zhang, Jiong-Tang Li

**Affiliations:** Key Laboratory of Aquatic Genomics, Ministry of Agriculture and Rural Affairs, Beijing Key Laboratory of Fishery Biotechnology, Chinese Academy of Fishery Sciences, Beijing 100141, China

**Keywords:** common carp, fatty acid elongase 5, poly-unsaturated fatty acids, duplication, dominant expression

## Abstract

Most diploid freshwater and marine fish encode one *elovl5* elongase, having substrate specificity and activities towards C18, C20 and C22 polyunsaturated fatty acids (PUFAs). The allo-tetraploid common carp is hypothesized to encode two duplicated *elovl5* genes. How these two *elovl5* genes adapt to coordinate the PUFA biosynthesis through elongase function and expression divergence requires elucidation. In this study, we obtained the full-length cDNA sequences of two *elovl5* genes in common carp, named as *elovl5a* and *elovl5b*. Functional characterization showed that both enzymes had elongase activity towards C18, C20 and C22 PUFAs. Especially, the activities of these two enzymes towards C22 PUFAs ranged from 3.87% to 8.24%, higher than those in most freshwater and marine fish. The Elovl5a had higher elongase activities than Elovl5b towards seven substrates. The spatial-temporal expression showed that both genes co-transcribed in all tissues and development stages. However, the expression levels of *elovl5b* were significantly higher than those of *elovl5a* in all examined conditions, suggesting that *elovl5b* would be the dominantly expressed gene. These two genes had different potential transcriptional binding sites. These results revealed the complicated roles of *elovl5* on PUFA synthesis in common carp. The data also increased the knowledge of co-ordination between two homoeologs of the polyploid fish through function and expression divergence.

## 1. Introduction

Long-chain polyunsaturated fatty acids (LC-PUFAs) are essential compounds that play key roles in numerous metabolic and physiological processes, which ensure normal cellular function [1,2,3]. The biosynthesis of LC-PUFAs in vertebrates involves consecutive desaturation and elongation reactions that convert the fatty acids (FAs) of C18:3n-3 (α-linolenic acid) and C18:2n-6 (linoleic acid) to PUFAs of longer chains [4]. The products include some essential nutrients, for instance, ARA (C20:4n-6) [5], EPA (C20:5n-3) [6] and DHA (C22:6n-3) [7]. The LC-PUFA biosynthesis involves the activities of the elongases of very long-chain fatty acid (Elovl), which add two carbon molecules into the end of the carbon chain of the FA substrate [8]. Fish are a major source of LC-PUFA for human diets [9]. Furthermore, LC-PUFAs were the necessary nutrients for fish growth. Identifying the LC-PUFAs biosynthesis-related genes would benefit the selection breeding to improve the LC-PUFA contents in the cultured fish. Increasing LC-PUFA contents in the cultured fish may provide more LC-PUFAs for humans but also decrease the requirement of LC-PUFAs in the aquaculture feed.

The Elovl5 elongase, one of the most important elongases, catalyzes the rate-limiting reaction of elongating PUFAs. Most diploid fish encoded one *elovl5*, having versatile substrate specificity and activities towards C18 (C18:2n-6, C18:3n-3, C18:4n-3 and C18:3n-6), C20 (C20:5n-3 and C20:4n-6) and C22 PUFAs (C22:4n-6 and C22:5n-3). The *elovl5* genes in the polyploid fish were reported to have two different evolutionary fates. The auto-tetraploid Atlantic salmon encodes two duplicated *elovl5* genes, *elovl5a* and *elovl5b*. Both genes lost the elongation activities towards C18:3n-3 and C18:2n-6 and had extremely low conversion rates of C22:5n-3 and C22:4n-6 (1%). Furthermore, the Elovl5b had higher elongase activities towards C18:3n-6, C20:5n-3 and C20:4n-6 than Elovl5a [10]. In the auto-tetraploid rainbow trout, theoretically, two duplicated *elovl5* genes should exist, while only one was retained after the whole genome duplication [11]. This *elovl5* was active with C18–20 PUFA substrates but not with C22 PUFAs. Whether there is another function scenario of duplicated *elovl5* genes in the polyploid fish has not been answered yet. In the auto-tetraploid Atlantic salmon, the homeologous *elovl5a* and *elovl5b* genes had differential and asymmetrical expression patterns. Both genes had higher expression levels in the liver, intestine and brain than other tissues. However, *elovl5a* over-expressed in the intestine compared to *elovl5b*, while the trend was reversed in the liver. Likewise, little is known about other expression divergence patterns of duplicated *elovl5* genes in the polyploidy fish.

Common carp, a world-wide cultured species, underwent a very recent allo-tetraploidization event and performed parallel subgenome evolution with divergent expression processes [12]. This allo-tetraploid genome has a slow gene loss rate and encodes almost twice gene numbers compared with the diploid fish. The homeologs had high nucleotide identities (>90%). However, it is still unknown whether the functions of two homeologs were identical. This fish is able to convert the dietary C18 PUFAs to LC-PUFAs [13] and are predicted to encode two *elovl5* genes. However, whether they have identical substrate specificity and elongase activities is still unknown. In addition, studying their expression processes and the related promoter activities would explore novel expression divergence patterns of the duplicated *elovl5* genes in the polyploid fish.

To study the functions and expression patterns of common carp *elovl5* genes, we obtained the full-length sequences of two *elovl5* genes. Functional characterization studies including the heterologous expression of both enzymes in yeasts and molecular docking were performed. Their expression patterns were determined using quantitative real-time PCR (qPCR) and whole mount in situ hybridization. Finally, the promoter activity assays were performed to identify the core promoter of each gene. These data exhibit novel functions and expression patterns of duplicated genes in the polyploid fish.

## 2. Results

### 2.1. The Structure Difference between Common Carp Elovl5a and Elovl5b

The full-length mRNA sequences of two common carp *elovl5* genes were obtained. One mRNA (GenBank accession number MK893918.1) had a length of 2834 bp and was aligned to the A13 chromosome of the common carp genome (from 811,708 bp to 835,469 bp). This gene was defined as *elvol5a* since it was located in the A subgenome of common carp. Its coding region started from 148 bp to 1023 bp of the mRNA and encoded a protein with a size of 291 amino acids. The other mRNA with a length of 2698 bp (GenBank accession number MK893919.2) was located in the B13 chromosome of the B subgenome (from 5,221,394 to 5,232,990 bp). It was termed as *elovl5b*—encoding a protein with the same size of 291 amino acids. The 5′UTR ended at 457 bp and the 3′UTR started from 1334 bp.

The allo-tetraploidization of common carp is hypothesized to be hybridized from two different progenitors [14]. Therefore, it is reasonable that their gene structures were much different. The genomic region of *elovl5a* was longer than *elovl5b*. Even though they were both comprised of eight exons and seven introns, the total intronic length of *elovl5a* was longer than *elovl5b* (Supplementary Figure S1). These two genes also had different lengths of 5′UTR and 3′UTR. Using the *elovl5b* mRNA as the subject, the global identity of the *elovl5a* mRNA was 86.96% with a coverage of 71%. The identities and sequence coverages of 5‘UTR (80.56% and 96%) and 3′UTR (84% and 76.57%) between two homoeologs were lower than those of CDS (94.75% and 100%), respectively, suggesting that the UTRs were less conserved than the CDS.

The phylogenetic analysis clustered the tetraploid Cyprinidae *elovl5a* (*Cyprinus carpio*, *Carassius auratus* and *Sinocyclocheilus anshuiensis*) into one group. The tetraploid Cyprinidae *elovl5b* were grouped together with the *Barbonymus gonionotus elovl5* into another cluster, suggesting that the common carp *elovl5b* might originate from the diploid Barbinae fish *elovl5*. These two groups were then clustered with *elovl5* from the other fish (Supplementary Figure S2). These data supported the allo-tetraploidization event in common carp. In Atlantic salmon, *elovl5a* and *elovl5b* were clustered together and then grouped with the northern pike *elovl5*, indicating the auto-tetraploidization in salmon.

### 2.2. Dominant Elongase Activities of Elovl5a Compared with Elovle5b

The protein sequences of Elovl5a and Elovl5b were 89% and 91% identical to zebrafish Elovl5 sequence (GenBank accession number NP_956747.1), respectively. The zebrafish Elovl5 had elongase activities towards C18–22 PUFAs [15]. Due to the high identities, did two common carp Elovl5 have the same functions as zebrafish Elovl5? The protein identity between common carp Elovl5a and Elovl5b was 94.5% with only 15 mutations (Supplementary Figure S3). Whether these two genes had identical elongation activities has not been answered. To avoid the possible contaminates from the vector, we examined the FA profile of the yeasts, which were transformed with the empty vectors and grown with the substrate of C18:2n-6. No product peak was found in the profile, demonstrating no conversion ability of the pYES2 vector. Then, the yeasts transformed with the recombinant pYES2 vectors containing common carp *elovl5a* and *elovl5b* cDNA inserts (pYES2-elovl5a and pYES2-elovl5b) were grown in the presence of the potential substrates. Eight substrates included C18 (C18:2n-6, C18:3n-3, C18:3n-6 and C18:4n-3), C20 (C20:4n-6 and C20:5n-3) and C22 (C22:4n-6 and C22:5n-3), respectively. The FA profiles in these yeasts were used to determine the substrate specificities and elongase activities of these two enzymes.

**Figure 1:**
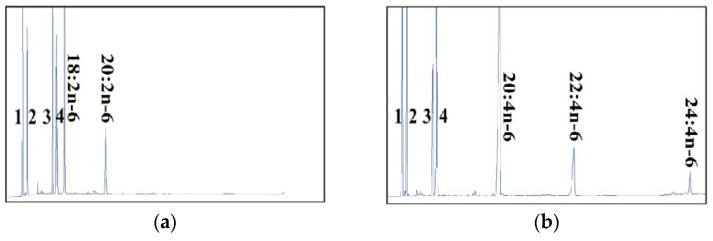

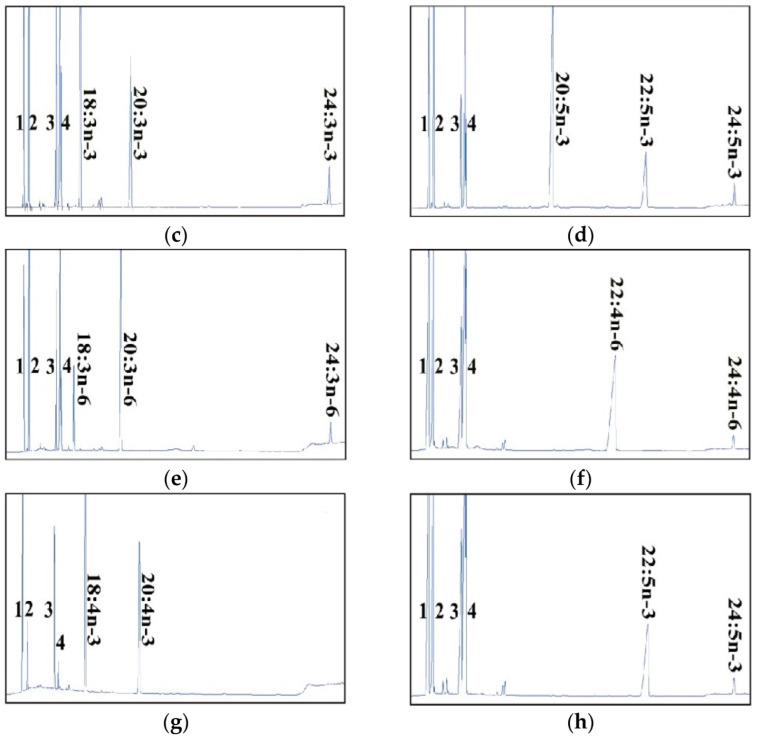
The elongase activities of common carp Elovl5a towards different substrates in the transgenic yeast grown with (**a**) C18:2n-6, (**b**) C20:4n-6, (**c**) C18:3n-3, (**d**) C20:5n-3, (**e**) C18:3n-6, (**f**) C22:4n-6, (**g**) C18:4n-3 and (**h**) C22:5n-3. The peaks from 1 to 4 represent C16:0 (1), C16:1n-7 (2), C18:0 (3) and C18:1n-9 (4), respectively, which are the four main endogenous FAs of yeasts. The substrates and their corresponding products were indicated. The vertical axis displayed the flame-ionization detector response peak. The horizontal axis showed the peak retention time (min).

The common carp Elovl5a had activities towards eight FA substrates including C18–22 PUFAs in the n-3 and n-6 pathways (Figure 1). It had the highest activities towards C18:3n-6, followed by C18:3n-3 and C18:4n-3. The elongase activities towards C20 PUFAs were lower than C18 PUFAs. The common carp Elovl5a had catalyzation activities towards C22:4n-6 and C22:5n-3 (Table 1, 4.66% and 8.24%, respectively).

**Figure 2:**
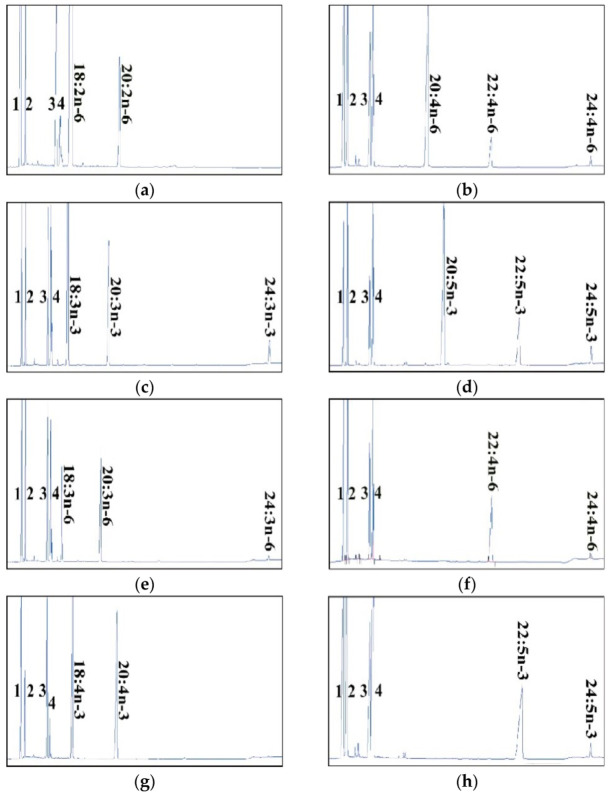
Elongase activities of the common carp Elovl5b in the transgenic yeasts grown in the presence of one substrate, including C18:2n-6 (**a**), C20:4n-6 (**b**), C18:3n-3 (**c**), C20:5n-3 (**d**), C18:3n-6 (**e**), C22:4n-6 (**f**), C18:4n-3 (**g**) and C22:5n-3 (**h**). The peaks from 1 to 4 represent the four main endogenous FAs of yeasts, including C16:0 (1), C16:1n-7 (2), C18:0 (3) and C18:1n-9 (4). The substrates and their corresponding elongated products are indicated. The vertical axis displays the flame-ionization detector response peak. The horizontal axis shows the peak retention time (min).

The common carp Elovl5b also had the elongase activities towards the same eight types of substrates as Elovl5a (Figure 2). Likewise, the activities of Elovl5b towards C18 PUFAs were the highest, followed by C20 and C22 PUFAs. Except C20:5n-3, the conversion rates of Elovl5a towards the other seven substrates were higher than Elovl5b, suggesting higher elongase activities of Elovl5a. In general, the average conversion rate of Elovl5a (31.3%) was higher than Elovl5b (25.8%, Table 1).

### 2.3. In Silico Molecular Docking Difference between Elovl5a and Elovl5b

Due to no homologous structure template available for modeling, the models of Elovl5a and Elovl5b were predicted using the I-TASSER program [16]. Since Elovl5a and Elovl5b had a protein identity of 94.5%, it is reasonable that their structures were almost identical (Supplementary Figure S4A). Their binding pockets were predicted to be hydrophobic. The average cavity volumes of Elovl5a and Elovl5b for eight PUFAs were 326.6 Å3 and 276.9 Å3, respectively (Supplementary Figure S4B,C).

In general, in a larger binding pocket, one substrate had more interaction with the amino acids as the binding force increased, as represented by a lower docking energy [17]. The average docking energy of eight substrates with Elovl5a (−8.23 kcal/mol) was lower than the one with Elovl5b (−7.82 kcal/mol, Supplementary Table S1), indicating a higher binding force of the former than the latter towards the same substrates. This trend was consistent with higher elongase activity of Elovl5a than Elovl5b.

### 2.4. Higher Expression Levels of Elovl5b Than Elovl5a

**Figure 3:**
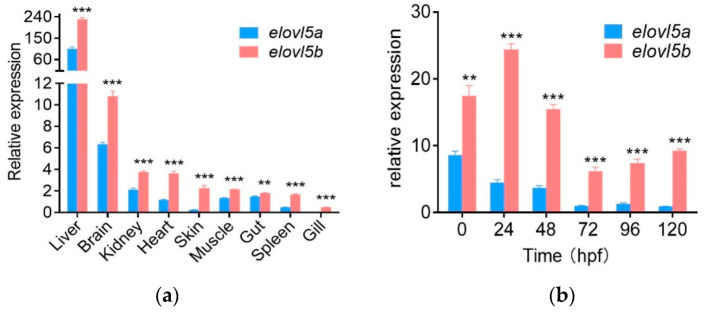
The expression levels of elovl5a and elovl5b in mature common carp tissues. (**a**) The expression levels across nine tissues. (**b**) The levels across six developmental stages from 0 to 120 h post-fertilization (hpf). The results are shown as the mean ± standard deviation (SD). Two asterisks refer to the *p* value < 0.01 and three asterisks refer to the *p* value < 0.001.

The expression levels of *elovl5a* and *elovl5b* across nine tissues in the mature common carp were analyzed with qPCR (Figure 3a). Both genes were co-expressed in all tissues analyzed, indicating that they might co-function in the same tissues. The highest levels of these two genes were both found in liver, similar to the tissue expression patterns of *elovl5* in other fish [10]. The brain, kidney and heart also showed high expression levels of these genes. The lowest expression levels of these two genes were observed in gill. In all tissues, *elovl5b* had significantly higher expression levels than *elovl5a*, suggesting that the former was the dominant gene in the PUFA. Intriguingly, these two genes exhibited a significantly positive expression pattern (correlation value = 0.8, one-tailed *p* value = 0.005). All these data suggested that they exhibited a dominant but correlated spatial expression.

Temporal expression levels of *elvol5a* and *elovl5b* were studied with qPCR on cDNA samples obtained at six developmental stages from 0 to 120 hpf (Figure 3b). They were expressed at 0 hpf and throughout embryonic development. They were co-expressed in the same stages, suggesting their co-functions during development. In all stages, *elovl5b* had significantly higher expression levels than *elovl5a*, suggesting that the former was the dominantly expressed gene during development. These six stages were divided into two phases, before hatching (BH, 0–48 hpf) and after hatching (AH, 72–120 hpf). The transcript levels of these two genes at BH were noticeably higher than those at AH. We also observed the significantly positive correlation between *elovl5a* and *elovl5b* during development (correlation value = 0.771, one-tailed *p* value = 0.036). All these data suggested that they exhibited a divergent but correlated temporal expression pattern.

**Figure 4:**
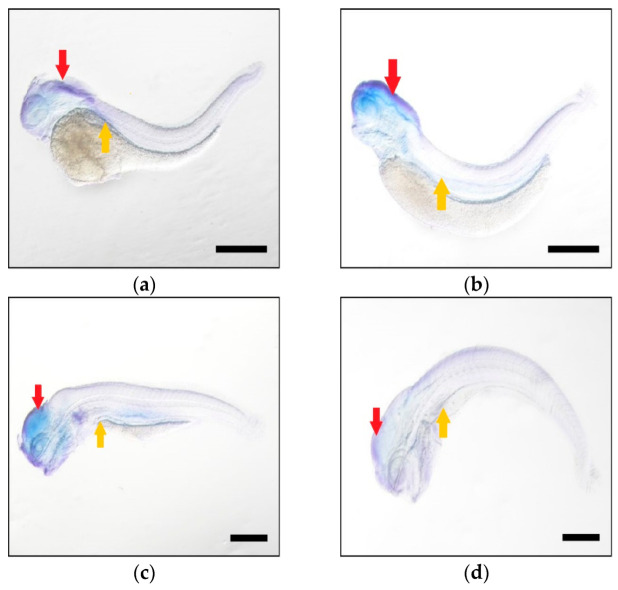
The tissue expressions of *elovl5a* during common carp larvae detected using WISH at 48 hpf (**a**), 72 hpf (**b**), 96 hpf (**c**) and 120 hpf (**d**). Larvae were hybridized with the probes and the blue dye represented the expression signal. Black scale bars: 500 μm. The red arrow indicated the head region and the duct was marked with the yellow arrow.

**Figure 5:**
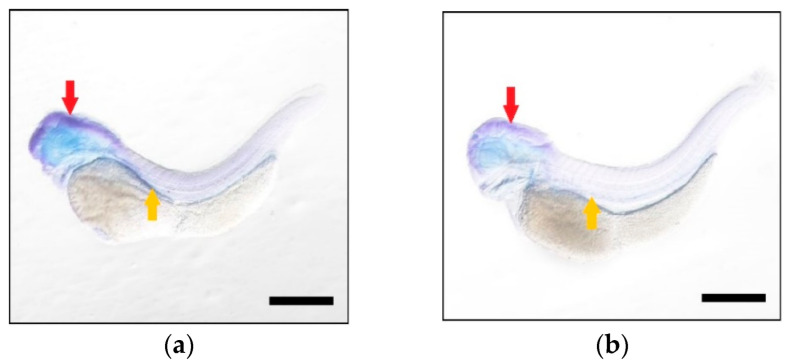

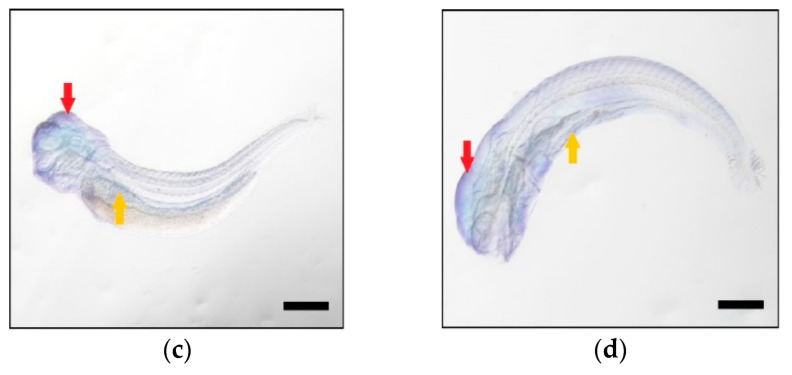
The tissue expressions of *elovl5b* during common carp larvae development detected using WISH at 48 hpf (**a**), 72 hpf (**b**), 96 hpf (**c**) and 120 hpf (**d**). Larvae were hybridized with the probes and the blue dye represented the expression signal. Black scale bars: 500 μm. The red arrow indicated the head region and the duct was marked with the yellow arrow.

To examine the spatial expressions of these two genes during embryo development, whole mount in situ hybridization (WISH) was performed at four stages (48, 72, 96 and 120 hpf, Figure 4 and Figure 5). The yolks were absorbed and almost disappeared at 120 hpf. At four times, both genes were observed in head and duct, suggesting that they co-transcribed in the same developmental tissues. For each gene, the signal in head was higher than the other regions, indicating that these two homeologous had the same dominantly expressed tissue during development.

### 2.5. Correlation between the Larvae PUFA Contents and the Expressions of Two Genes

To further investigate the correlation between the expressions of *elovl5a* and *elovl5b* and the FA contents during early development, the FA contents of common carp embryos and larvae at the above six developmental stages were analyzed. At each developmental stage, the saturated FAs were the most, followed by the PUFAs. The monounsaturated FAs were the fewest (Supplementary Table S2). The content ratio between the n-6 PUFAs and n-3 PUFAs ranged from 2.35 to 3.38, indicating that the n-6 PUFAs were the main forms of PUFAs. From 24 hpf to 120 hpf, the total contents of the saturated FAs and n-3 PUFAs increased by 38.8% and 7.7%, respectively. However, the contents of the monounsaturated FAs and n-6 PUFAs decreased by 42.2% and 20.2%, respectively. Among the n-6 PUFAs, C18:2n-6 and C20:4n-6 were the two main forms. The former content decreased while the latter content was stable. Among the n-3 PUFAs, DHA (C22:6n-3) accounted for the majority, ranging from 83.9% to 92%.

The maternal mRNAs maintain in the embryos to sustain development after fertilization [18]. The fed diet contains the exogenous PUFAs, influencing the measurement of endogenous PUFAs generated by elongases. Therefore, to study the effects of the expression levels of larvae *elovl5a* and *elovl5b* on the contents of the endogenous PUFA products, we first estimated the contents of C18 PUFA substrates (18:3n-3 and 18:2n-6) and the contents of the products from 72 hpf to 120 hpf (Table 2). The content of C18:2n-6, the total content of the elovl5-related n-6 PUFA products, and the total content of elovl5-related n-6 PUFAs continually decreased as development proceeded (Supplementary Table S2). The decreases might be resulted from the consumption. However, the ratio between the n-6 product and C18:2n-6 increased, suggesting the elevated n-6 elongation processes. Likewise, the content of C18:3n-3, the total content of the elovl5-related n-3 PUFA products and the total content of the elovl5-related n-3 PUFAs continually decreased. We also observed the increasing trend of the ratio between the n-3 products and C18:3n-3. This trend also hinted the elevated n-3 elongation processes.

From 72 to 120 hpf, the expression level of *elovl5b* had a significantly positive correlation with the ratio between the n-6 products and C18:2n-6 (correlation = 0.993, one-tailed *p* value = 0.036). The level was also significantly positively correlated with the ratio between the n-3 products and C18:3n-3 (correlation = 0.995, one-tailed *p* value = 0.033). These data suggested that the increasing expression of *elovl5b* might contribute to the elevated n-6 and n-3 elongation processes. In contrast, the expression level of *elovl5a* had no significant correlation with the above two ratios (C18:2n-6, correlation = −0.353, one-tailed *p* value = 0.385; C18:3n-3, correlation = −0.341, one-tailed *p* value = 0.389). Hence, the expression of *elovl5a* might have had less contributions to the elevated n-6 and n-3 elongation processes than *elovl5b*.

### 2.6. Promoter Activity Analysis of Elovl5a and Elovl5b

**Figure 6:**
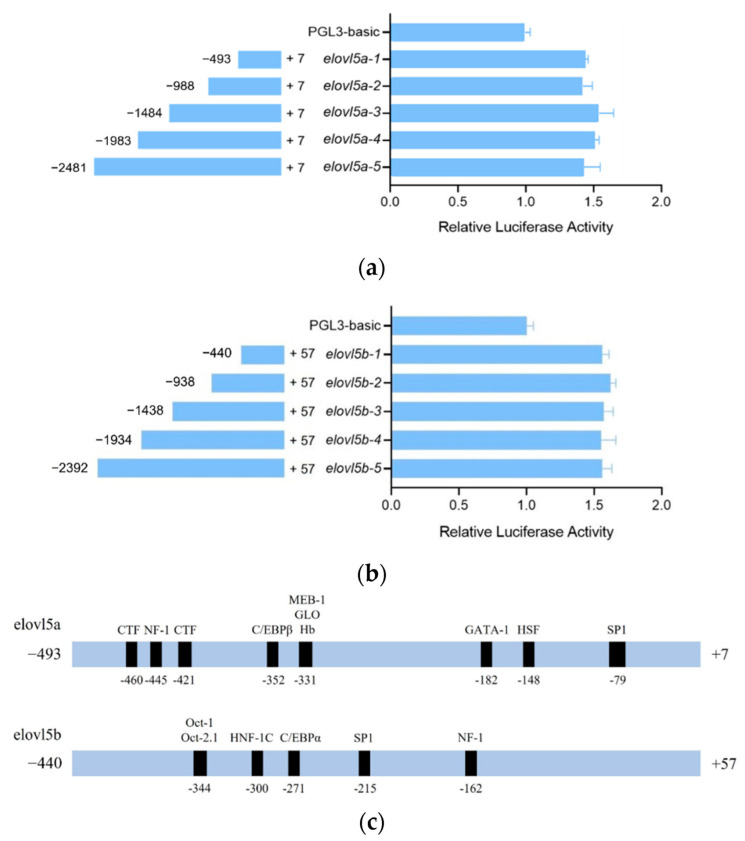
Promoter activity analysis of common carp elovl5a (**a**) and elovl5b (**b**). The results are reported as the mean ± SD. (**c**) The predicted TF binding sites in the core promoters of elovl5a and elovl5b. Many TFs had close binding sites.

We finally examined whether the promoter activities between the two genes were different. A full-length potential promoter region of *elovl5a* and four truncated regions were constructed with the pGL3-basic luciferase reporter vector. The promoter activities of the five regions of each gene were higher than the pGL3-basic vector without any insert (Figure 6). The five regions of *elovl5a* showed no significant difference of the luciferase activities (Figure 6a), suggesting that the core promoter of *elolv5a* started from the upstream 493 bp to the 7 bp in the 5′UTR. The full-length candidate region and four truncated regions of *elovl5b* also had no significant difference in the luciferase activities (Figure 6b). The core promoter of *elovl5b* might start from the upstream 440 bp to the 57 bp in the 5′UTR.

We predicted the potential transcriptional factor binding sites in the core promoters. The core promoters of these two genes had a low coverage of 22% with an identity of 89.8%. We identified the binding sites of ten transcriptional factors (TFs) in the core promoter of *elovl5a* (Figure 6c), where the binding sites of three TFs were close. Six potential TFs had binding sites in the core promoter of *elovl5b*, where two factors had close binding sites. Many reported that PUFA-related TFs, including the specific protein 1 (SP1) [19], hepatocyte nuclear factor-1 (HNF-1) [20], CCAAT enhancer binding protein (C/EBP) [21], and nuclear factor 1 (NF-1) [22], were identified in both core promoters. However, their locations were different. Furthermore, the numbers, types and locations of the other TF binding sites were also divergent. All results exhibited the differences of both the sequences and binding sites between the core promoters of these two genes.

## 3. Discussion

The fish *elovl5* genes were reported to have low or a loss of elongase activity towards C22 PUFAs in the n-3 and n-6 pathways [23]. The Elovl5 conversion rates of C22:5n-3 in zebrafish, catfish, tilapia, Atlantic salmon, turbot and sea bream, ranged from 0.6% to 4.9%. The conversion rates of C22:4n-6 in these fish were up to 0.9%. Grass carp, a Cyprinidae species close to common carp, was unable to convert C22 PUFAs [24]. We provided evidence that common carp Elovl5a and Elovl5b had higher elongation activities towards C22:5n-3 (8.24% and 6.0%) and C22:4n-6 (4.66% and 3.87%) than the above widely cultured fish and zebrafish. We demonstrated that both genes of common carp co-transcribed in all examined tissues and developmental stages. Therefore, it is speculated that the functions of these two homoeologs might be accumulative to increase the conversion rates of C22:5n-3 and C22:4n-6, improving the PUFA contents in common carp.

Using *elovl5a* and *elovl5b* as models, we identified the specific or common evolutional fates of duplicated homeologs in the polyploidy fish. Previously we summarized the divergent and versatile expression processes in common carp and goldfish [12]. We validated the co-transcription, dominant expression and strong expression correlation between common carp *elovl5a* and *elovl5b* in multiple tissues and developmental stages. Likewise, in Atlantic salmon, *elovl5a* and *elovl5b* had the features of co-transcription and dominant expression. However, these two salmon genes had a low expression correlation (0.41) [25].

With respect to the function evolutional fate, in Atlantic salmon, the duplicated *elovl5* genes had high elongase activities towards four substrates (Supplementary Table S3) and extremely low activities towards C22 PUFAs. They had no activities to C18:2n-6 and C18:3n-3. In rainbow trout, one of the duplicated *elovl5* genes was lost and the remaining one was active with C18–20 PUFA substrates but not with C22 PUFAs [11]. In common carp, both duplicated homeologs were retained and their elongase functions were also active with C18–22 PUFAs, representing the third function evolution fate. However, we also found in both allo-tetraploid and auto-tetraploid fish that there was one dominantly functioned gene in two homeologs. Interestingly, in this pair of common carp homeologs, the function dominance and expression dominance adhered to two different genes. The separation of function dominance and expression dominance on different genes was also observed in Atlantic salmon [25]. The separation improved the expression plasticity and functional flexibility, making contributions to the subgenome adaptation of the polyploid fish.

Elovl2 also participated in the elongation of PUFAs and was reported to have higher elongase activity towards C20 and C22 PUFAs than Elovl5 [11,26]. From searching the common carp reference genome with zebrafish Elovl2 (Genbank accession: NP_001035452.1), two duplicated *elovl2* genes were identified (XM_042714143.1 in the A24 chromosome and XM_042751565.1 in the B24 chromosome). The functions on the PUFA biosynthesis and the temporal–spatial expressions of the potential *elovl2a* and *elovl2b* will be studied in the future.

Recently, we scanned the polymorphisms in *elovl5a* and *elovl5b* [27]. Two non-synonymous-coding SNPs in *elovl5b*, E5b.172 and E5b.782 were significantly associated with several PUFA contents, while only one synonymous SNP in *elovl5a* was significantly associated with the C20:5n-3 PUFA content. The results suggested that *elovl5b* might be the major gene corresponding to common carp PUFA contents. In our study, *elovl5b* had higher expression levels than *elovl5a* in all examined conditions, further supporting this conclusion. This gene would be an important target for selection breeding to improve the PUFA contents in common carp.

## 4. Materials and Methods

### 4.1. Animals and Tissue Collection

Mature common carp samples (two years old, n = 10) were collected at the Fangshan experimental base of Chinese Academy of Fishery Science. Then, the fish were anaesthetized with Eugenol (Yuanye Bio-Technology, Shanghai, China) at a concentration of 10 mg/L. Nine tissues including the liver, brain, muscle, gut, spleen, skin, gills and kidney from each fish were collected, then frozen in liquid nitrogen and finally stored at −80 °C.

### 4.2. Cloning of Elovl5a and Elovl5b cDNAs

The total RNA of each common carp was isolated from each frozen tissue using Trizol (Thermo Scientific, Waltham, MA, USA) following manufacturer’s instruction We determined the concentration and integrity of the RNA by using the NanoDrop 2000 spectrophotometer (Thermo Scientific, Waltham, MA, USA) with 1% agarose gel electrophoresis. Then, the first strand cDNA was synthesized from total RNA using RevertAid First Strand cDNA Synthesis Kit (Thermo Scientific, Waltham, MA, USA). The cDNA was used for obtaining the full-length transcripts and measuring the spatial expression levels.

We predicted the duplicated common carp *elovl5* transcripts by aligning the zebrafish *elovl5* protein (GenBank: NP_956747.1) against the recent chromosome-quality common carp genome [12] using tblastn. Considering the allo-tetraploidization of common carp, we retained the top significant two genomic regions for the downstream rapid amplification of cDNA ends (RACEs). These two regions were located in the chromosomes of A13 and B13, respectively. The duplicated *elovl5* genes were denominated as *elovl5a* (in the A13 chromosome) and *elovl5b* (in the B13 chromosome), respectively.

First, to validate these two predicted transcripts, we designed the specific primers to amplify the transcripts (Supplementary Table S4). The PCR reactions for *elovl5a* and *elovl5b* included an initial denaturation of 94 °C for 4 min, followed by 30 cycles of 94 °C/20 s, 60 °C/20 s and 72 °C/1 min, and with a final extension step at 72 °C for 5 min (2 × EasyTaq SuperMix, Tiangen, Beijing, China). The PCR products were determined on 1% agarose gel and then purified with EasyPure Quick Gel Extraction Kit (TransGen Biotech, Beijing, China). Second, based on the validated transcripts, we performed the 5′ and 3′ RACEs with SMART 5′/3′ RACE Kit (Clontech, Mountain View, CA, USA). All the purified PCR products were sequenced with paired-end Sanger sequencing on an ABI 3730 XL machine (TaiheGene, Beijing, China).

### 4.3. Sequence and Phylogenetic Analysis

For each gene, all sequences were assembled with CAP3 [28] to a full-length transcript, the codon region of which was predicted with ORFfinder [29]. The full-length transcript was aligned to the reference genome and the gene structure was displayed with Gene Structure Display Server 2.0 [30]. Together with other vertebrate Elovl5 proteins (Supplementary Table S5), the common carp Elovl5a and Elovl5b proteins were aligned with Clustalw [31]. The multiple alignment was used to construct a phylogenetic tree using MEGA 7.0 software [32] with the Neighbor-Joining method [33] and 1000 bootstraps. The gene tree was displayed with Evolview v3 [34].

### 4.4. Functional Characterization of the Common Carp Elovl5a and Elovl5b in Yeast

The *elovl5a* and *elovl5b* CDS regions were amplified by the primers including the Hind III and Xho I enzyme restriction sites (Supplementary Table S6). The digested *elovl5a* and *elovl5b* CDS sequences with the restriction endonucleases Hind III and Xho I, were purified with the AxyPrep PCR Cleanup Kit (Corning, Corning, NY, USA). The pYES2.0 vector (Thermo Scientific, Waltham, MA, USA) was also digested with the same endonucleases. With T4 DNA ligase (Thermo Scientific, Waltham, MA, USA), the digested PCR products were linked to the digested vector. The recombinant plasmids (pYES2-elovl5a and pYES2-elovl5b) were transformed into *Saccharomyces cerevisiae*-competent cells with the S.c. EasyComp Transformation Kit (Thermo Scientific, Waltham, MA, USA), respectively. The recombinant yeasts were grown according to previously methods [4,35].

The yeasts were added with one of the following substrates: 0.5 mM C18:2n-6, 0.5 mM C18:3n-3, 0.5 mM C18:3n-6, 0.5 mM C18:4n-3, 0.75 mM C20:4n-6, 0.75 mM C20:5n-3, 1 mM C22:4n-6 and 1 mM C22:5n-3. After 48 h, the yeasts were collected by centrifugation at 5000× g for 10 min and then freeze-dried into the powders. The 100 mg powders were dissolved in 2 mL of 0.5 M NaOH-methanol using ultrasound for 10 min and heated at 100 °C for 30 min. The solution was added with 2 mL of 14% boron trifluoride-methanol solution and heated at 100 °C for 1 h. The fatty acid methyl esters (FAMEs) were extracted with 1 mL of hexane and 5 mL of saturated NaCl solution and then centrifuged at 10,000 rpm for 5 min. The top phase was transferred to a clean glass tube. The bottom phase was added with 1 mL hexane and a new top solution was extracted following the same strategy. These two FAME extracts were combined and dried with the oxygen-free nitrogen. The FAMEs were then resuspended with 1 mL of hexane and filtered through a nylon syringe filter SCAA-104 (ANPEL, Shanghai, China). The esters were finally analyzed by gas chromatography on an Agilent 7890A system (Agilent Technologies, Santa Clara, CA, USA) using nitrogen as the carrier gas, following our previous method [36]. The system was equipped with a capillary column (112-88A7, Agilent Technologies, internal diameter of 0.25 mm and length of 100 m), a flame ionization detector and a CTC analytics autosampler injector.

The retention time of all FAMEs in a Supelco 37 Component FAMEs standard mix (Nu-chek Prep, Inc., Elysian, MN, USA) was used as reference. We compared the retention times of each peak with the reference times to determine which FA the peak belonged to. We focused on the above eight types of substrates and their corresponding products (C20:2n-6, C20:3n-6, C22:4n-6, C24:4n-6, C24:3n-6, C20:3n-3, C20:4n-3, C22:5n-3, C24:3n-3 and C24:5n-3). To determine the elongase activities of Elovl5a and Elovl5b, we first calculate the total area of the substrate and its corresponding products. Second, the conversion rate from one substrate to one product was calculated as [the product area/(all products area + substrate area)] × 100, as described previously [4]. Finally, the conversion rate of one substrate by Elovl5a or Elovl5b was the total conversion rates of all corresponding products.

### 4.5. Structure Modelling and Docking of Elovl5a and Elovl5b

Since there was no available structure of the Elovl5 homolog in the RCSB PDB database [37] for homolog modelling, we performed ab initio modeling for Elovl5a and Elovl5b using the I-TASSAR program [16]. The structure with the best prediction score was further optimized with AMBER16 [38]. Three-dimensional (3D) structures of eight PUFA substrates were obtained from the Pubchem database [39] (Supplementary Table S1).

To measure the docking energy of one substrate PUFA to one enzyme, the docking between the enzyme model and the substrate was performed with AutoDock 4.2 [40]. Each docking was replicated at least 30 times. The docking result with the best energy was applied into the molecular dynamic (MD) simulations using AMBER16. The average structure of the simulations was used to analyze the interaction force.

### 4.6. Spatial-Temporal Expression of Elovl5a and Elovl5b

The expression levels of these two genes in nine tissues of mature common carp were measured with qPCR. The qPCR was carried out in triplicate on a 7500 Real Time PCR System (Thermo Scientific, Waltham, MA, USA). The reaction was performed in 15 µL of reaction volume containing 7.5 µL SYBR Green Realtime PCR Master Mix (TOYOBO, Osaka, Japan), 1 µL cDNA, 0.3 µL forward and reverse primers (Supplementary Table S7) and 5.9 µL nuclease-free water. The qPCR profiles contained an initial activation step at 95 °C for 2 min, followed by 40 cycles: 15 s at 95 °C, 15 s at 60 °C and 30 s at 72 °C. The product sizes were checked by agarose gel electrophoresis. The expression of the beta-actin gene was used as the reference to estimate the target gene expression levels with the 2^−∆∆Ct^ method [41]. All data were reported as mean ± SD. In each tissue, we performed an independent *t*-test to compare whether the expressions of these two genes were significantly different.

To study the expression levels of the target genes during the embryo and larvae development of common carp, we collected six pools of 100 embryos at 0, 24, 48, 72, 96 and 120 hpf, respectively. The total RNAs of each pool were extracted and reversely transcribed into cDNAs. The qPCR, expression calculation and comparisons were performed as described above.

The digoxygenin-labelled RNA probes of common carp *elovl5a* and *elovl5b* were in vitro synthesized. First, the specific primers (Supplementary Table S8) were used to amplify the different templates for synthesizing *elovl5a* and *elovl5b* antisense probes. The genomic DNA from common carp liver was used as the PCR template with EasyTaq PCR Supermix kit (TransGen Biotech, Beijing, China) following the manufacturer’s instructions. Second, the purified PCR products were linked into the pEASY-T3 vector (TransGen Biotech, Beijing, China) using TA cloning. The plasmid DNA in the positive clones was extracted with the plasmid DNA MiniPrep Kit (Thermo Scientific, Waltham, MA, USA). The M13 primers consisting of the T7 promoters were used to amplify the probe templates. Finally, the digoxygenin-labelled RNA probes were in vitro transcribed using MAXIscript™ T7 transcription kit (Thermo Scientific, Waltham, MA, USA).

We collected the common carp embryos at 48, 72, 96 and 120 hpf for WISH. The WISH with the digoxygenin-labelled probes of *elovl5a* and *elovl5b* were performed on these embryos as previously described [42,43].

### 4.7. Fatty Acid Analysis of Common Carp Embryos at Different Developmental Stages

For each pool of common carp embryos or larvae, the FAMEs preparation and FA identification were performed as described in Section 4.4. In each pool, we calculated the total area of the identified FAs. The relative content (RC) of each FA in each pool was calculated as [the FA area/total area] × 1000. The RC of the saturated FAs was the sum contents of C14:0, C15:0, C16:0, C18:0 and C20:0. Since the areas of C16:1n-9, C18:1n-7, C22:1 and C24:1n-9 were extremely small, the RC of the monounsaturated FAs was the sum contents of C16:1n-7, C18:1n-9 and C20:1. The RCs of C18:2n-6, C18:3n-6, C20:2n-6, C20:3n-6, C20:4n-6, C22:4n-6 and C22:5n-6 were summed up to represent the RC of n-6 PUFAs. The RC of n-3 PUFAs was the total content of C18:3n-3, C18:4n-3, C20:3n-3, C20:4n-3, C20:5n-3, C22:5n-3 and C22:6n-3. The RC of the total PUFA was the sum of the n-3 PUFA RC and the n-6 PUFA RC.

During the embryo development and before hatching, Elovl5 converted the maternal C18:2n-6 and C18:3n-3 into the PUFAs of longer chains. Therefore, we focused on the ratio between the elovl5-related products and substrates. The elovl5-related n-6 PUFAs included the substrate (C18:2n-6) and the products (C20:2n-6, C20:3n-6 and C22:4n-6). The n-6 product/substrate ratio was the RC of the products divided by one of the substrates. The elovl5-related n-3 PUFAs included the substrate (C18:3n-3) and the products (C20:3n-3, C20:4n-3 and C22:5n-3). The n-3 product/substrate ratio was the RC of the products divided by the one of the C18:3n-3.

### 4.8. Identifying the Core Promoters of Common Carp Elovl5a and Elovl5b

To identify the core promoter region of *elovl5a*, we designed five forward primers (elovl5a-2.5k-F, elovl5a-2k-F, elovl5a-1.5k-F, elovl5a-1k-F and elolvl5a-0.5k-F) having a Nhe I digestion site, and a common reverse primer (elovl5a-R) with a Xho I site (Supplementary Table S9) to generate a full-length promoter fragment (elovl5a-2.5k, 2488 bp) and four truncated fragments: (i) elovl5a-2k (1990 bp); (ii) elovl5a-1.5k (1491 bp); (iii) elovl5a-1k (995 bp); and (iv) elovl5a-0.5k (500 bp). The amplifications were initiated with denaturation at 94 °C for 4 min; 35 cycles of denaturation at 94 °C for 20s; annealing at 60 °C for 20s; extension at 72 °C for 2 min 30 s (2.5k), 2 min (2k), 1 min 30 s (1.5k), 1 min (1k), 30 s (0.5k); and final extension at 72 °C for 5 min. The products of different lengths were purified by AxyPrep^TM^ PCR Cleanup Kit (Axygen Biosciences, Tewksbury, MA, USA) and then digested with Nhe I and Xho I, respectively. The pGL3-basic vector (Promega, Madison, WI, USA) was also digested with the same endonucleases. The digested five fragments were linked to the vector with T4 DNA ligase (Takara, Osaka, Japan). The recombinant plasmids were extracted using EndoFree Plasmid Giga Kit (Tiangen, Beijing, China) and confirmed by sequencing, as described above. Likewise, we also generated five recombinant plasmids containing the *elovl5b* promoter regions of different lengths.

The common carp EPC cells were incubated in the M199 medium (Thermo Scientific, Waltham, MA, USA) with 10% fetal bovine serum (FBS) (Thermo Scientific, Waltham, MA, USA) at 28 °C without CO_2_. The cells were seeded in a 96-well plate before transfection. Transient transfections were performed with Lipofectamine 2000™ reagent (Invitrogen, USA) in the presence of Opti-MEM medium (Thermo Scientific, Waltham, MA, USA) when a 90–95% confluence was reached. A total of 1 µg of the pGL3 recombinant vectors and 0.1 µg of the pRL-CMV vector (Promega Corporation, Madison, WI, USA) were co-transfected into the cells, which were incubated at 28 °C.

At 48 h after transfection, the EPC cells in each experiment group were collected and then transferred into the M199 medium mixed with the same amount of the dual luciferase reagent (Promega, Madison, WI, USA). The firefly luciferase activity value of cell lysates was measured with a 96-well microplate-reading luminometer (BioTek, Friedrichshall, Germany). After adding the Stop & Glo reagent in sequence, the renilla luciferase activity value was measured. The measurements were performed in triplicate. The relative promoter activity in one group was calculated as the ratio of these two values.

For each gene, we compared the activity of each promoter region to determine the core promoter. With AliBaba (version 2.1, available online: http://www.gene-regulation.com/, accessed date: 9 May 2019) and the default parameters, we predicted the potential transcriptional factors and their binding sites in the core promoter of each gene.

## 5. Conclusions

Common carp is a widely cultured allo-tetraploid species. Studying the functions and expressions of two homeologs, *elovl5a* and *elovl5b* not only explored the abilities of common carp generating the PUFAs but also revealed the co-ordination between two homoeologues of the polyploid fish through function and expression divergence. We demonstrated that both Elovl5a and Elovl5b had the elongase activities towards C18, C20, and C22 n-3 and n-6 PUFAs. Even the activities of common carp Elovl5a and Elovl5b towards C22 PUFAs were higher than Elovl5b in other widely cultured fish. We observed the separation of function dominance and expression dominance into two different genes, suggesting the coordination of two duplicated genes for the adaptation to the PUFA requirement.

## Figures and Tables

**Table 1:** Functional characterization of Elovl5a and Elovl5b.

FA Substrate	Product	Elovl5a Conversion (%)	Elovl5b Conversion (%)	Activity
C18:2n-6	C20:2n-6	11.63	8.42	C18→C20
C18:3n-6	C20:3n-6	74.24	69.63	C18→C20
	C24:3n-6	8.45	1.14	C22→C24
	Total	82.69	70.74	
C18:4n-3	C20:4n-3	44.91	43.91	C18→C20
C18:3n-3	C20:3n-3	36	22.97	C18→C20
	C24:3n-3	9.2	3.9	C22→C24
	Total	45.2	26.87	
C20:5n-3	C22:5n-3	25.12	26.52	C20→C22
	C24:5n-3	4.83	3.68	C22→C24
	Total	29.95	30.2	
C20:4n-6	C22:4n-6	17.90	13.29	C20→C22
	C24:4n-6	5.02	2.92	C22→C24
	Total	22.92	16.21	
C22:4n-6	C24:4n-6	4.66	3.87	C22→C24
C22:5n-3	C24:5n-3	8.24	6.00	C22→C24

The conversion rate was expressed as a percentage of one product in the total exogenous FAs. The total conversion rate of one substrate was the sum of the rates converted to different products.

**Table 2:** The contents of elovl5-related PUFAs during common carp embryo development.

FA Substrates	0 hpf	24 hpf	48 hpf	72 hpf	96 hpf	120 hpf
C18:2n-6	121.22	108.9	104.32	85.53	68.28	37.92
C20:2n-6	13.26	11.67	11.9	10.99	10.39	7.91
C20:3n-6	75.96	19.89	19.49	17.58	16.17	11.6
C22:4n-6	12.3	11.26	10.87	10.18	10.42	8.12
elovl5-related n-6 PUFAs	222.74	151.72	146.58	124.28	105.26	65.55
total n-6 PUFA products	101.52	42.82	42.26	38.75	36.98	27.63
n-6 products/substrate ratio	0.84	0.39	0.405	0.453	0.54	0.73
C18:3n-3	5.71	5.06	4.56	3.06	2.08	1.06
C20:3n-3	0.94	1.08	1.02	0.85	0.83	0.71
C20:4n-3	0.59	0.47	0.48	0.4	0.37	0.17
C22:5n-3	1.47	3.7	3.54	3.86	3.65	3.04
elovl5-related n-3 PUFAs	8.71	10.31	9.6	8.17	6.93	4.98
total n-3 PUFA products	3	5.25	5.04	5.11	4.85	3.92
n-3 products/substrate ratio	0.525	1.04	1.11	1.67	2.33	3.70

Each number of one FA represented its relative content to all FAs. The content of the elovl5-related n-6 PUFAs was the total content of C18:2n-6, C20:2n-6, C20:3n-6 and C22:4n-6. The content of the total n-6 PUFA products was the sum of C20:2n-6, C20:3n-6 and C22:4n-6. The ratio of the n-6 products/substrate was equal to the content of the total n-6 PUFA products divided by the one of C18:2n-6. The content of the elovl5-related n-3 PUFAs was the total content of C18:3n-3, C20:3n-3, C20:4n-3 and C22:5n-3. The content of the total n-3 PUFA products was the sum of C20:3n-3, C20:4n-3 and C22:5n-3. The ratio of n-3 products/substrate was equal to the content of the total n-3 PUFA products divided by the one of C18:3n-3.

## Data Availability

The data presented in this study are available in Supplementary Material.

## Supplementary Materials

The following supporting information can be downloaded at: https://www.mdpi.com/article/10.3390/ijms232314666/s1.

## References

[1] Gow R.V., Hibbeln J.R. (2014). Omega-3 fatty acid and nutrient deficits in adverse neurodevelopment and childhood behaviors. Child Adolesc. Psychiatr. Clin. N. Am..

[2] Lands B. (2017). Highly unsaturated fatty acids (HUFA) mediate and monitor food’s impact on health. Prostaglandins Other Lipid Mediat..

[3] Rennison J.H., Van Wagoner D.R. (2009). Impact of dietary fatty acids on cardiac arrhythmogenesis. Circ. Arrhythm. Electrophysiol..

[4] Li Y., Monroig O., Zhang L., Wang S., Zheng X., Dick J.R., You C., Tocher D.R. (2010). Vertebrate fatty acyl desaturase with Δ4 activity. Proc. Natl. Acad. Sci. USA.

[5] Davis-Bruno K., Tassinari M.S. (2011). Essential fatty acid supplementation of DHA and ARA and effects on neurodevelopment across animal species: A review of the literature. Birth Defects Res. Part B Dev. Reprod. Toxicol..

[6] Roussel C., Anunciação Braga Guebara S., Plante P.L., Desjardins Y., Di Marzo V., Silvestri C. (2022). Short-term supplementation with ω-3 polyunsaturated fatty acids modulates primarily mucolytic species from the gut luminal mucin niche in a human fermentation system. Gut Microbes.

[7] Sun G.Y., Simonyi A., Fritsche K.L., Chuang D.Y., Hannink M., Gu Z., Greenlief C.M., Yao J.K., Lee J.C., Beversdorf D.Q. (2018). Docosahexaenoic acid (DHA): An essential nutrient and a nutraceutical for brain health and diseases. Prostaglandins Leukot. Essent. Fat. Acids.

[8] Jakobsson A., Westerberg R., Jacobsson A. (2006). Fatty acid elongases in mammals: Their regulation and roles in metabolism. Prog. Lipid Res..

[9] Guo F., Bunn S.E., Brett M.T., Kainz M.J. (2017). Polyunsaturated fatty acids in stream food webs—High dissimilarity among producers and consumers. Freshw. Biol..

[10] Carmona-Antoñanzas G., Tocher D.R., Taggart J.B., Leaver M.J. (2013). An evolutionary perspective on Elovl5 fatty acid elongase: Comparison of Northern pike and duplicated paralogs from Atlantic salmon. BMC Evol. Biol..

[11] Gregory M.K., James M.J. (2014). Rainbow trout (Oncorhynchus mykiss) Elovl5 and Elovl2 differ in selectivity for elongation of omega-3 docosapentaenoic acid. Biochim. Biophys. Acta (BBA)-Mol. Cell Biol. Lipids.

[12] Li J.T., Wang Q., Huang Yang M.D., Li Q.S., Cui M.S., Dong Z.J., Wang H.W., Yu J.H., Zhao Y.J., Yang C.R. (2021). Parallel subgenome structure and divergent expression evolution of allo-tetraploid common carp and goldfish. Nat. Genet..

[13] Castro L.F., Tocher D.R., Monroig O. (2016). Long-chain polyunsaturated fatty acid biosynthesis in chordates: Insights into the evolution of Fads and Elovl gene repertoire. Prog. Lipid Res..

[14] Ma W., Zhu Z.H., Bi X.Y., Murphy R.W., Wang S.Y., Gao Y., Xiao H., Zhang Y.P., Luo J. (2014). Allopolyploidization is not so simple: Evidence from the origin of the tribe Cyprinini (Teleostei: Cypriniformes). Curr. Mol. Med..

[15] Agaba M., Tocher D.R., Dickson C.A., Dick J.R., Teale A.J. (2004). Zebrafish cDNA encoding multifunctional Fatty Acid elongase involved in production of eicosapentaenoic (20:5n-3) and docosahexaenoic (22:6n-3) acids. Mar. Biotechnol..

[16] Roy A., Kucukural A., Zhang Y. (2010). I-TASSER: A unified platform for automated protein structure and function prediction. Nat. Protoc..

[17] Stank A., Kokh D.B., Fuller J.C., Wade R.C. (2016). Protein Binding Pocket Dynamics. Acc. Chem. Res..

[18] Dworkin M.B., Dworkin-Rastl E. (1990). Functions of maternal mRNA in early development. Mol. Reprod. Dev..

[19] Wolf S.S., Roder K.H., Schweizer M. (1998). The general transcription factor Sp1 plays an important role in the regulation of fatty acid synthase. Biochem. Soc. Trans..

[20] Wang X., Hassan W., Zhao J., Bakht S., Nie Y., Wang Y., Pang Q., Huang Z. (2019). The impact of hepatocyte nuclear factor-1α on liver malignancies and cell stemness with metabolic consequences. Stem Cell Res. Ther..

[21] Sabatier M., Birsen R., Lauture L., Dehairs J., Angelino P., Mouche S., Heiblig M., Boet E., Sahal A., Saland E. (2022). C/EBPα confers dependence to fatty acid anabolic pathways and vulnerability to lipid oxidative stress in FLT3-mutant leukemia. bioRxiv.

[22] Dong Y., Wang S., Chen J., Zhang Q., Liu Y., You C., Monroig Ó., Tocher D.R., Li Y. (2016). Hepatocyte Nuclear Factor 4α (HNF4α) Is a Transcription Factor of Vertebrate Fatty Acyl Desaturase Gene as Identified in Marine Teleost Siganus canaliculatus. PLoS ONE.

[23] Agaba M.K., Tocher D.R., Zheng X., Dickson C.A., Dick J.R., Teale A.J. (2005). Cloning and functional characterisation of polyunsaturated fatty acid elongases of marine and freshwater teleost fish. Comp. Biochem. Physiol. Part B Biochem. Mol. Biol..

[24] Marrero M., Monroig Ó., Navarro J.C., Ribes-Navarro A., Pérez J.A., Galindo A., Rodríguez C. (2022). Metabolic and molecular evidence for long-chain PUFA biosynthesis capacity in the grass carp Ctenopharyngodon idella. Comp. Biochem. Physiol. Part A Mol. Integr. Physiol..

[25] Morais S., Monroig O., Zheng X., Leaver M.J., Tocher D.R. (2009). Highly unsaturated fatty acid synthesis in Atlantic salmon: Characterization of ELOVL5- and ELOVL2-like elongases. Mar. Biotechnol..

[26] Liu C., Ye D., Wang H., He M., Sun Y. (2020). Elovl2 But Not Elovl5 Is Essential for the Biosynthesis of Docosahexaenoic Acid (DHA) in Zebrafish: Insight from a Comparative Gene Knockout Study. Mar. Biotechnol..

[27] Zhang Y., Li Q.-S., Ye Y.-Q., Wang Q., Sun X.-Q., Zhao R., Li J.-T. (2022). Association Analysis between Genetic Variants of *elovl5a* and *elovl5b* and Poly-Unsaturated Fatty Acids in Common Carp (*Cyprinus carpio*). Biology.

[28] Huang X., Madan A. (1999). CAP3: A DNA sequence assembly program. Genome Res..

[29] Wheeler D.L., Chappey C., Lash A.E., Leipe D.D., Madden T.L., Schuler G.D., Tatusova T.A., Rapp B.A. (2000). Database resources of the National Center for Biotechnology Information. Nucleic Acids Res..

[30] Hu B., Jin J., Guo A.Y., Zhang H., Luo J., Gao G. (2015). GSDS 2.0: An upgraded gene feature visualization server. Bioinformatics.

[31] Larkin M.A., Blackshields G., Brown N.P., Chenna R., McGettigan P.A., McWilliam H., Valentin F., Wallace I.M., Wilm A., Lopez R. (2007). Clustal W and Clustal X version 2.0. Bioinformatics.

[32] Tamura K., Stecher G., Kumar S. (2021). MEGA11: Molecular Evolutionary Genetics Analysis Version 11. Mol. Biol. Evol..

[33] Saitou N., Nei M. (1987). The neighbor-joining method: A new method for reconstructing phylogenetic trees. Mol. Biol. Evol..

[34] Subramanian B., Gao S., Lercher M.J., Hu S., Chen W.H. (2019). Evolview v3: A webserver for visualization, annotation, and management of phylogenetic trees. Nucleic Acids Res..

[35] Zhu K.C., Song L., Guo H.Y., Guo L., Zhang N., Liu B.S., Jiang S.G., Zhang D.C. (2018). Identification of Fatty Acid Desaturase 6 in Golden Pompano Trachinotus Ovatus (Linnaeus 1758) and Its Regulation by the PPARαb Transcription Factor. Int. J. Mol. Sci..

[36] Zhang Y., Sun X.-Q., Ye Y.-Q., Wang Q., Li Q.-S., Zhao R., Wang H.-W., Li J.-T. (2021). Association between the Polymorphisms of *fads2a* and *fads2b* and Poly-Unsaturated Fatty Acids in Common Carp (*Cyprinus carpio*). Animals.

[37] Burley S.K., Bhikadiya C., Bi C., Bittrich S., Chen L., Crichlow G.V., Christie C.H., Dalenberg K., Di Costanzo L., Duarte J.M. (2021). RCSB Protein Data Bank: Powerful new tools for exploring 3D structures of biological macromolecules for basic and applied research and education in fundamental biology, biomedicine, biotechnology, bioengineering and energy sciences. Nucleic Acids Res..

[38] Case D., Betz R., Cerutti D.S., Cheatham T., Darden T., Duke R., Giese T.J., Gohlke H., Götz A., Homeyer N. (2016). Amber 16, University of California, San Francisco.

[39] Kim S., Thiessen P.A., Bolton E.E., Chen J., Fu G., Gindulyte A., Han L., He J., He S., Shoemaker B.A. (2016). PubChem Substance and Compound databases. Nucleic Acids Res..

[40] Morris G.M., Huey R., Lindstrom W., Sanner M.F., Belew R.K., Goodsell D.S., Olson A.J. (2009). AutoDock4 and AutoDockTools4: Automated docking with selective receptor flexibility. J. Comput. Chem..

[41] Rao X., Huang X., Zhou Z., Lin X. (2013). An improvement of the 2ˆ(-delta delta CT) method for quantitative real-time polymerase chain reaction data analysis. Biostat. Bioinform. Biomath..

[42] Cunningham R.L., Monk K.R. (2018). Whole Mount In Situ Hybridization and Immunohistochemistry for Zebrafish Larvae. Methods Mol. Biol..

[43] Thisse B., Thisse C. (2014). In situ hybridization on whole-mount zebrafish embryos and young larvae. Methods Mol. Biol..

