# Supplementary material for: Dominant Elongase Activity of Elovl5a but Higher Expression of Elovl5b in Common Carp (Cyprinus carpio)
Source: Int J Mol Sci. 2022 Nov 24;23(23):14666. doi: 10.3390/ijms232314666 (PMC9741273; doi:10.3390/ijms232314666)
Supplement: Supplementary file 1 [file ijms-23-14666-s001.zip › ijms-1977979-supplementary.pdf]

Supplementary Figure S1. Gene structures of zebrafish *elolv5*, common carp *elovl5a*, and *elovl5b*.

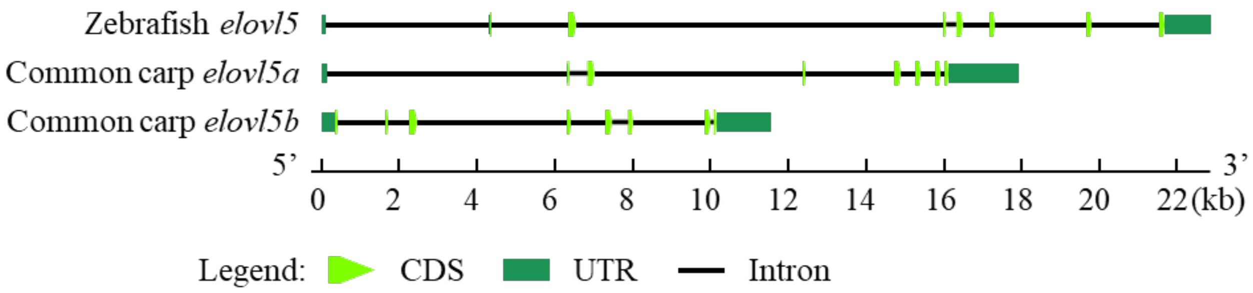

The CDS regions were indicated with light green boxes. The UTR regions were indicated with dark green boxes. The introns were indicated with black lines. The gene structures were illustrated with Gene Structure Display Server (GSDS) 2.0 [1].

Supplementary Figure S2. Phylogenetic tree of vertebrate Elovl5 family

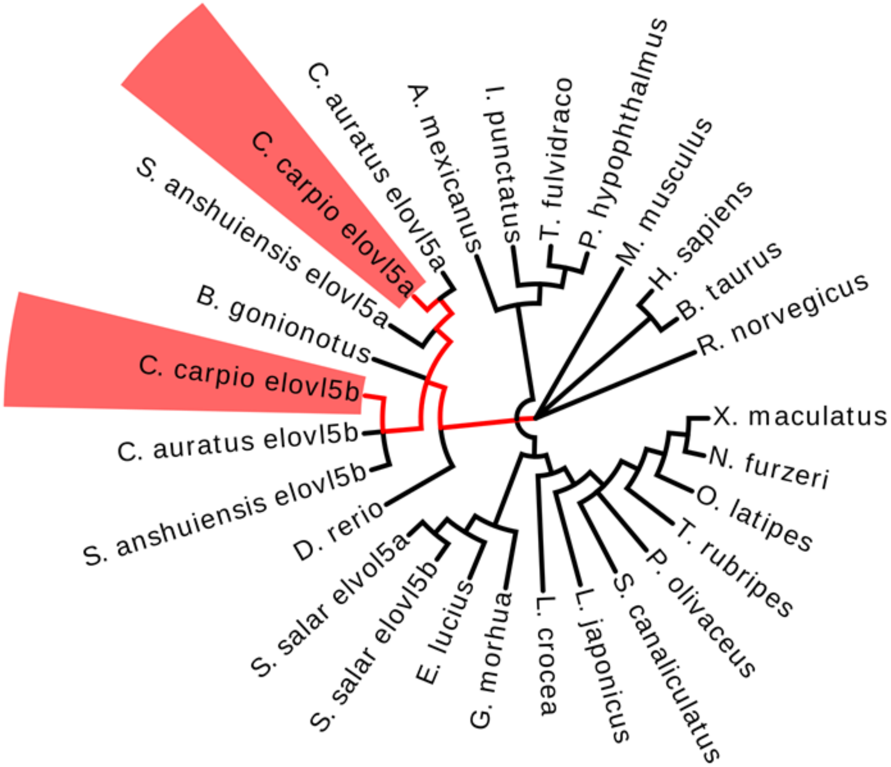

Comparing the protein sequences of common carp Elovl5a and Elovl5b with other vertebrate Elovl5 proteins.

Supplementary Figure S3. Comparing the protein sequences of common carp Elovl5a and Elovl5b

```
                10        20        30        40        50
        ....|....|....|....|....|....|....|....|....|....|
Elovl5a METFNHKVNTYIDSWMGPRDPRVRGWLLLDNYIPTFAFTVMYLLIVWMGP
Elovl5b MEAFNHRVNTYIDSWMGPRDPRVRGWLLLDNYIPTFAFTVMYLLVVWMGP

                60        70        80        90       100
        ....|....|....|....|....|....|....|....|....|....|
Elovl5a KYMKNRQPYSCRALLVPYNLGLTLLSFYMFYELVMSVYQGRYNFFCQNTH
Elovl5b KYMKNRQPYSCRALLVPYNLGLTLLSLYMFYELVMSVYQGGYNFFCQNTH

               110       120       130       140       150
        ....|....|....|....|....|....|....|....|....|....|
Elovl5a SGGEADNRMINVLWWYYFSKLIEFMDTFFFILRKNNHQITFLHVYHHATM
Elovl5b SGGEADNRMMNVLWWYYFSKLIEFMDTFFFILRKNNHQITFLHVYHHATM

               160       170       180       190       200
        ....|....|....|....|....|....|....|....|....|....|
Elovl5a LNIWWFVMNWVPCGHSYFGSTFNSFIHVLMYSYYGLSAVPAMRPYLWWKK
Elovl5b LNIWWFVMNWVPCGHSYFGATFNSFIHVLMYSYYGLSAVPALRPYLWWKK

               210       220       230       240       250
        ....|....|....|....|....|....|....|....|....|....|
Elovl5a YITQGQLVQFVLTMFQTSCAVVWPCGFPMGWLYFQITYMITLILLFSNFY
Elovl5b YITQGQLVQFVLTMFQTSCAVVWPCGFPMGWLYFQITYMITLILLFTNFY

               260       270       280       290
        ....|....|....|....|....|....|....|....|.
Elovl5a IKTYKSHAGSRKSGLSNGSINSHTNGVTSNEKVKYRKPRTD
Elovl5b IKTYKRHAGSRKTDYSNGSINGHTNGVASNEKVKYRKPRAD
```

The protein sequences were aligned with Clustal [2]. The red letters represented different amino acids between two proteins.

Supplementary Figure S4. The modeling of common carp Elovl5a and Elovl5b

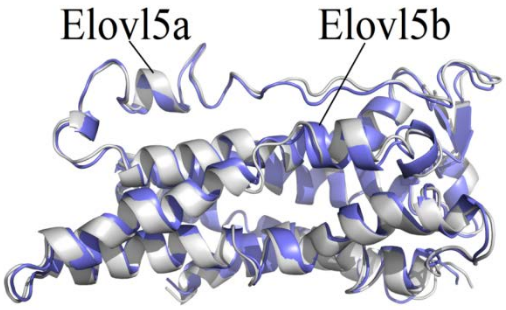

(**a**)

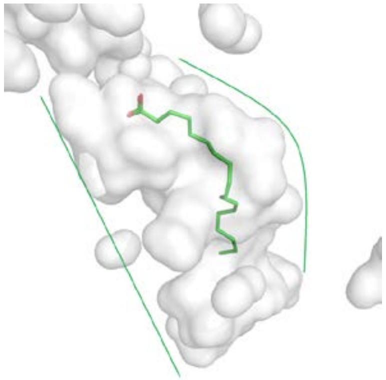

(**b**)

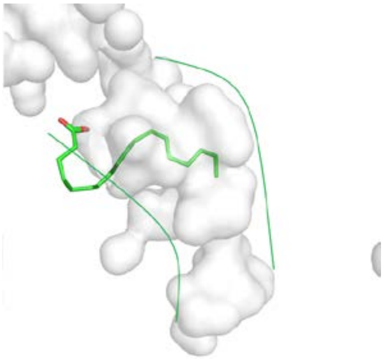

(**c**)

(a) The superimposed graph of the three dimensional structures of Elovl5a (white) and Elovl5b (purple). These two proteins had almost identical structures. The pockets of Elovl5a (b) and Elovl5b (c) proteins. The green chains represented the PUFA substrates.

Supplementary Table S1. Molecular docking energy of Elovl5a and Elovl5b

**Substrate**		**Docking energy (kcal/mol)**	
	Pubchem ID	Elovl5a	Elovl5b
18:2n-6	5280450	-8.07	-7.09
18:3n-3	5280934	-8.20	-7.72
18:3n-6	5280933	-7.97	-7.98
18:4n-3	5312508	-8.18	-8.37
20:4n-6	444899	-8.06	-8.81
20:5n-3	446284	-8.34	-8.27
22:5n-3	5497182	-8.48	-7.77
22:4n-6	5497181	-8.53	-6.52

Supplementary Table S2. FA contents of common carp embryos and larvae at six developmental stages.

Fatty acids	0 hpf	24 hpf	48 hpf	72 hpf	96 hpf	120 hpf
C14:0	4.45±0.02	4.20±0.01	4.20±0.05	4.10±0.01	3.75±0.01	3.80±0.03
C15:0	1.40±0.10	1.90±0.02	2.11±0.02	2.12±0.02	2.41±0.04	2.89±0.07
C16:0	289.69±3.30	284.47±0.14	293.16±2.26	317.53±0.60	317.39±0.49	351.78±0.62
C18:0	201.59±6.11	95.75±3.13	94.90±1.12	122.35±0.47	124.07±0.11	176.47±0.47
C20:0	0.30±0.01	0.48±0.01	0.91±0.02	1.14±0.01	1.31±0.02	1.91±0.01
Total saturated	497.44±2.72	386.80±3.05	395.28±3.43	447.23±0.99	448.93±0.41	536.86±0.87
C16:1n-7	10.86±0.86	18.13±0.09	15.89±1.51	1.35±0.10	12.53±0.18	8.20±0.19
C18:1n-9	111.85±7.16	205.33±3.06	202.84±3.16	184.63±0.38	167.34±0.10	121.08±0.28
C20:1	11.88±0.06	11.39±0.01	11.44±0.01	9.90±0.07	9.16±0.09	6.55±0.07
Total monounsaturated	134.59±6.24	234.85±3.16	230.17±4.67	195.88±0.50	189.03±0.16	135.82±0.14
C18:2n-6	121.22±0.42	108.90±0.05	104.32±0.53	85.53±0.21	68.28±0.19	37.92±0.06
C18:3n-6	6.02±0.17	10.37±0.05	16.92±0.38	33.96±1.17	39.20±0.32	46.39±0.61
C20:2n-6	13.26±0.16	11.67±0.04	11.90±0.06	10.99±0.05	10.39±0.05	7.91±0.04
C20:3n-6	75.96±8.99	19.89±0.09	19.49±0.07	17.58±0.07	16.17±0.11	11.60±0.03
C20:4n-6	23.82±0.03	103.05±0.08	101.01±0.26	96.30±0.17	102.36±0.34	96.04±0.18
C22:4n-6	12.30±0.65	11.26±0.05	10.87±0.07	10.18±0.11	10.42±0.05	8.12±0.35
C22:5n-6	21.37±0.53	22.73±0.07	21.92±0.07	21.04±0.14	22.85±0.11	21.85±0.25
Total n-6 PUFA	273.97±8.21	287.86±0.17	286.42±0.93	275.56±0.10	269.66±0.51	229.83±0.19
C18:3n-3	5.71±0.02	5.06±0.06	4.56±0.30	3.06±0.01	2.08±0.01	1.06±0.13
C18:4n-3	0.63±0.08	0.61±0.01	0.54±0.01	0.44±0.03	0.41±0.01	0.27±0.01
C20:3n-3	0.94±0.42	1.08±0.01	1.02±0.02	0.85±0.05	0.83±0.11	0.71±0.03
C20:4n-3	0.59±0.04	0.47±0.01	0.48±0.06	0.40±0.05	0.37±0.03	0.17±0.03
C20:5n-3	4.02±0.02	3.63±0.01	3.53±0.01	2.84±0.05	2.91±0.04	2.53±0.08
C22:5n-3	1.47±0.08	3.70±0.02	3.54±0.09	3.86±0.38	3.65±0.19	3.04±0.10
C22:6n-3	80.65±4.08	75.96±0.27	74.47±0.16	69.97±0.50	82.14±0.33	89.70±0.78
Total n-3 PUFA	94.01±4.68	90.51±0.28	88.13±0.43	81.42±0.55	92.39±0.23	97.48±0.78
Total PUFA	367.98±3.53	378.37±0.17	374.55±1.36	356.98±1.45	362.04±0.49	327.31±0.93

Each number represented the relative content of one FA to all FAs.

Supplementary Table S3. The elongase activity differences of homoeologues in allo-tetraploid common carp and auto-tetraploid Atlantic salmon

	**Common carp**			**Atlantic salmon#**		
FA Substrate	Elovl5a	Elovl5b	Difference (Elovl5a-Elovl5b)	Elovl5a	Elovl5b	Difference (Elovl5b-Elovl5a)
C18:2n-6	11.63	8.42	3.21	-	-	-
C18:3n-6	82.69	70.74	11.95	48	71	23
C18:4n-3	44.91	43.91	1	63	62	-1
C18:3n-3	45.2	26.87	18.33	-	-	-
C20:5n-3	29.95	30.2	-0.25	37	69	32
C20:4n-6	22.92	16.21	6.71	24	49	25
C22:4n-6	4.66	3.87	0.79	1	1	0
C22:5n-3	8.24	6.00	2.24	1	1	0

the data were cited from [3].

Supplementary Table S4. Primers for RACE amplification of common carp *elovl5a* and *elovl5b*

**Primers**	**Sequence (5'~3')**
5'RACE-*elovl5a*	GACTGTTTATTGATCCATTCGAGAG
5'RACE-*elovl5b*	CGTTCGAGTAATCGGTCTTCC
3'RACE-*elovl5a*-p1#	CCACAGTATCTCCAGCCTGC
3'RACE-*elovl5a*-p2*	GGGAATGAGCAGTTGGACCT
3'RACE-*elovl5b*-p1	TTCCCCCAAATAGCCCTGTG
3'RACE-*elovl5b*-p2	CATGTCTGGCAGTGAAGGGT
elovl5a validate-F&	ATGTCTACCATCACGCCACC
elovl5a validate-R	CTTGCTACACTGGGATGGCT
elovl5b validate-F	GGGGTGGCATCCAATGAGAA
elovl5b validate-R	CGAGGGAGGTGGTGTTACAG

The first round of primer used in the 3'RACE; * The second round of primer; & The primers used to validate the predicted transcripts.

Supplementary Table S5. Genbank accessions of the proteins used in the phylogenetic analysis**.**

Species	Genbank accession
Cyprinus carpio elovl5a	QGT76594.1
Cyprinus carpio elovl5b	QHB13631.1
Carassius auratus elovl5a	XP_026134883.1
Carassius auratus elovl5b	XP_026080513.1
Danio rerio	NP_956747.1
Homo sapiens	NP_001229757.1
Mus musculus	NP_599016.2
Bos taurus	NP_001040062.1
Rattus norvegicus	NP_599209.1
Paralichthys olivaceus	XP_019943235.1
Astyanax mexicanus	XP_022540762.1
Salmo salar elovl5a	AAO13175.2
Salmo salar elovl5b	NP_001130024.1
Lateolabrax japonicus	AVT42108.1
Esox Lucius	NP_001297915
Sinocyclocheilus anshuiensis elovl5a	XP_016310826.1
Sinocyclocheilus anshuiensis elovl5b	XP_016342043.1
Larimichthys crocea	NP_001290303.1
Pangasianodon hypophthalmus	XP_026795022.1
Xiphophorus maculatus	XP_005811937.1
Oryzias latipes	XP_004077464.1
Astyanax mexicanus	XP_022540762.1
Gadus morhua	AIG21333.1
Nothobranchius furzeri	SBP45045.1
Ictalurus punctatus	XP_017318183.1
Takifugu rubripes	XP_003964216.1
Tachysurus fulvidraco	XP_027021480.1
Siganus canaliculatus	ADE34561.1
Barbonymus gonionotus	AXG50646

Petromyzon marinus	ALZ50286.1

Supplementary Table S6. CDS amplification primers for insertion into yeast expression vector

Primers	Sequence (5'~3')	Restriction site
elovl5a-F	CCCAAGCTTACTAAAGGTTGAAGATGGAGACCT	Hind Ⅲ
elovl5a-R	CCGCTCGAGGAAGTGTCGTCAATCTGTGCG	Xho I
elovl5b-F	CCCAAGCTTAGGTTGGAGATGGAGGCCTTT	Hind Ⅲ
elovl5b-R	CCGCTCGAGCGAAGTGTCGTCAATCTGCG	Xho I
pYES2.0 screen-F*	TCTGGGGTAATTAATCAGCGA	
pYES2.0 screen-R	GCGTACACGCGTCTGTACAG	

The restriction sites were underlined in the primers. * the primers were used to screen the positive clones.

Supplementary Table S7. Primers for qPCR

Primers	Sequence (5'~3')
elovl5a-qPCR-F	TGTCTACCATCACGCCACCATG
elovl5a-qPCR-R	CCACATGGCCAAACTACAGCAC
elovl5b-qPCR-F	ACTGAGCACAGAAGGAAACA
elovl5b-qPCR-R	AGTTGTCCAGTAGAAGCCAT
β-actin-F	TGCAAAGCCGGATTCGCTGG
β-actin-R	AGTTGGTGACAATACCGTGC

Supplementary Table S8. Primers for WISH probe amplification

Primers	Sequence (5'~3')
WISH-*elovl5a*-F	TCACCACGCTACAGCCATTT
WISH-*elovl5a*-R	AAGCTCGCTCGATTAGTTCCA
WISH-*elovl5b*-F	ATTCCCCCAAATAGCCCTGTG
WISH-*elovl5b*-R	AAGAACTGTGCGCATACCCT
M13-F	GTAAAACGACGGCCAGT
M13-R	CAGGAAACAGCTATGAC

Supplementary Table S9. Primers to amplify the promoters of different sizes

Primers	Sequence (5'~3')	Restriction site
elovl5a-2.5K-F	CTAGCTAGCCACAAGTGCGACAGAATCGC	Nhe I
elovl5a-2K-F	CTAGCTAGCTGCTTTGCAGATTCTTTTGGCA	Nhe I
elovl5a-1.5K-F	CTAGCTAGCTGCTGCTTGCGTGTAATGTT	Nhe I
elovl5a-1K-F	CTAGCTAGCACACTGCATTACAGGTCAGC	Nhe I
elovl5a-0.5K-F	CTAGCTAGCGTGAGCACGTTTGGTGGAAC	Nhe I
elovl5a-promoter-R	CCGCTCGAGACTCGCTTTATACTGCCGCT	Xho I
elovl5b-2.5K-F	CTAGCTAGCCAGGCAAACTAGTTCACTCACT	Nhe I
elovl5b-2K-F	CTAGCTAGCCCGGAACTTATCGGAGACCC	Nhe I
elovl5b-1.5K-F	CTAGCTAGCGCAGCACTGACTGAACCCAT	Nhe I
elovl5b-1K-F	CTAGCTAGC CAAGTACAAGCACATGCCCC	Nhe I
elovl5b-0.5K-F	CTAGCTAGCCTAAGATGTGCAGTGGTTGGC	Nhe I
elovl5b-promoter-R	CCGCTCGAGTCAACGTAACTGTTTGACTTGC	Xho I

The restriction sites were underlined in the primers.

References

1. Hu, B.; Jin, J.; Guo, A.Y.; Zhang, H.; Luo, J.; Gao, G. GSDS 2.0: an upgraded gene feature visualization server. *Bioinformatics* **2015**, *31*, 1296-1297, doi:10.1093/bioinformatics/btu817.

2. Larkin, M.A.; Blackshields, G.; Brown, N.P.; Chenna, R.; McGettigan, P.A.; McWilliam, H.; Valentin, F.; Wallace, I.M.; Wilm, A.; Lopez, R.; et al. Clustal W and Clustal X version 2.0. *Bioinformatics* **2007**, *23*, 2947-2948, doi:10.1093/bioinformatics/btm404.

3. Morais, S.; Monroig, O.; Zheng, X.; Leaver, M.J.; Tocher, D.R. Highly unsaturated fatty acid synthesis in Atlantic salmon: characterization of ELOVL5- and ELOVL2-like elongases. *Marine biotechnology (New York, N.Y.)* **2009**, *11*, 627-639, doi:10.1007/s10126-009-9179-0.
